# La investigación en Atención Primaria: La ciencia de la proximidad - ¿Cómo transformar la práctica diaria en un motor para el avance científico?

**DOI:** 10.23938/ASSN.1177

**Published:** 2026-08-18

**Authors:** Nuria Goñi-Ruiz, Sara Furtado-Eraso

**Affiliations:** 1 Servicio Navarro de Salud-Osasunbidea. Gerencia de Atención Primaria. Navarra. España. Servicio Navarro de Salud-Osasunbidea Gerencia de Atención Primaria Navarra España; 2 Instituto de Investigación Sanitaria de Navarra (IdiSNA). Pamplona, Navarra. España. Instituto de Investigación Sanitaria de Navarra (IdiSNA) Pamplona Navarra España; 3 Universidad Pública de Navarra (UPNA). Departamento de Ciencias de la Salud. Pamplona, Navarra. España. Universidad Pública de Navarra (UPNA) Departamento de Ciencias de la Salud Pamplona Navarra España; 4 Red de Investigación en Cronicidad, Atención Primaria y Promoción de la Salud (RICAPPS). Pamplona. España. Red de Investigación en Cronicidad, Atención Primaria y Promoción de la Salud (RICAPPS) Pamplona España

El estudio que se publica en este número 49(2) de la revista Anales del Sistema Sanitario de Navarra sobre la prevalencia del consumo de cannabis en la población atendida en los centros de salud^1^ es mucho más que una cifra estadística; es un ejemplo claro de cómo la investigación en Atención Primaria (AP) actúa como un *sensor* privilegiado de nuestra realidad sanitaria. Al abrir el foco de investigación a grupos que a menudo quedan *huérfanos* de actividades preventivas, como es el caso de los adolescentes, la AP demuestra su capacidad auténtica para transformar la práctica clínica diaria y visibilizar necesidades que otros niveles asistenciales no llegan a detectar.

Este tipo de investigaciones, al igual que otras publicadas recientemente en esta revista^2^^,^^3^, ofrecen la oportunidad de reflexionar sobre la situación de la investigación en AP, abrir la mirada sobre su potencial y su valor intrínseco, y visibilizar su capacidad de poner a las personas en el centro de la investigación, permitiendo dar voz a las necesidad y prioridades sentidas de los y las pacientes, su entorno y su comunidad.

En este editorial reflexionamos sobre la evolución y la situación de la investigación en AP, sobre sus barreras (ciertamente reales) y también sobre sus fortalezas y su potencial (con frecuencia infravalorados).

## El valor de investigar en la *trinchera*

Aunque la investigación en AP en España ha mostrado en las últimas décadas un crecimiento notable y una creciente internacionalización^4^, persiste una tradicional brecha respecto del ámbito hospitalario que sigue siendo una realidad. Las barreras son reales y conocidas^5^: una elevada presión asistencial, la falta de tiempo reservado, las deficiencias estructurales, la escasez de líneas de investigación multicéntricas, la dispersión geográfica de los equipos que dificulta la constitución de grupos de investigación, la escasez de incentivos, la falta de formación metodológica, y la pérdida de motivación. Estas constituyen las barreras identificadas para la investigación en AP^5^^,^^6^.

Además, existen dos factores mantenidos en el tiempo que, aunque menos visibles, también determinan estas diferencias. En primer lugar, la escasa presencia de la AP en los entornos universitarios contribuye a transmitir a generaciones sucesivas de profesionales la idea implícita de que la investigación rigurosa y de calidad sucede en el hospital, infravalorando el potencial de la AP en la capacidad de generar conocimiento científico^7^. En segundo lugar, si bien los programas de formación sanitaria especializada en España recogen la investigación como competencia transversal, todavía queda recorrido para que esta sea una realidad que repercuta en la práctica asistencial.

Consecuencia de todo lo expuesto, y aunque el aumento de la producción científica muestra que existe capacidad e interés creciente en investigar en AP, persiste un problema de contexto, de tradición y de prioridades institucionales^5^.

La AP posee unas fortalezas y un potencial investigador que conviene poner en valor con la misma insistencia con la que se señalan sus dificultades:

- El *ojo clínico* de los y las profesionales de AP no solo aplica la evidencia científica, sino que también la impulsa. Cada observación clínica puede convertirse en una nueva pregunta de investigación. Además, el ámbito de trabajo de AP, marcado por la incertidumbre, con información fragmentada -y en el que es necesario tomar decisiones en condiciones reales (alejadas de los escenarios controlados del ensayo clínico)- propicia generar preguntas de investigación originales y pertinentes que contribuyen a una investigación clínica *útil*^8^ con capacidad real de transformar la práctica asistencial.
- La longitudinalidad, la proximidad y el vínculo que se establece con los y las pacientes en AP facilitan el reclutamiento, el seguimiento a largo plazo (uno de los mayores retos metodológicos de cualquier estudio prospectivo) y la convierten en un entorno idóneo para la investigación que no se centra únicamente en la enfermedad, sino también en los procesos de salud, los ciclos vitales y los determinantes que influyen en el continuo salud-enfermedad^9^^,^^10^. El seguimiento continuado de las personas permite analizar la evolución de las enfermedades a lo largo del tiempo, evaluar los efectos tardíos en ocasiones invisibilizados de los tratamientos, estudiar las secuelas persistentes de determinados procesos, y conocer la situación real de supervivientes de enfermedades como el cáncer.

## El poder del dato y la fuerza de la unión

Hoy en día, la AP es también un *motor de medicina de precisión*. Los registros clínicos de nuestra práctica diaria (diagnósticos, cuidados, prescripciones, resultados analíticos, actividades preventivas, derivaciones) constituyen un activo de valor incalculable. Herramientas como la base de datos BIFAP^11^, que sigue a cerca del 40% de la población española, o la vigilancia epidemiológica a través de las redes centinela, son fundamentales para tomar decisiones de salud pública basadas en el *mundo real*.

El fortalecimiento de una *investigación epidemiológica* a través de las redes centinela de AP es clave para la salud pública actual. Los registros clínicos en consulta permiten una vigilancia epidemiológica activa y basada en la comunidad, fundamental para la detección y el seguimiento de enfermedades transmisibles y no transmisibles. Tal como puso de manifiesto la pandemia por COVID^12^, investigar a partir de estos registros sirve para anticipar brotes, evaluar la efectividad de las vacunas en el mundo real y guiar la toma de decisiones sanitarias oportunas.

La AP, dispersa por definición, encuentra su mayor potencia investigadora precisamente en el trabajo en red. La creación en 2003 de la Red de Investigación en Actividades Preventivas y Promoción de la Salud (redIAPP) y su transformación en 2021 en la Red de Investigación en Cronicidad, Atención Primaria y Promoción de la Salud (RICAPPS), integrada en el programa estatal de Redes de Investigación Cooperativa Orientadas a Resultados de Salud (RICORS)^13^ del Instituto de Salud Carlos III, constituyen la prueba de que esta capacidad de red es real y competitiva a escala estatal e internacional.

Esta investigación colaborativa hace posible la *co-creación y el pensamiento colectivo*, favoreciendo esas sinergias que hacen posible un conocimiento más rico y cercano a la realidad, con menos sesgos individuales y una mayor capacidad para abordar problemas complejos y generar respuestas de mayor impacto. La colaboración entre equipos permite alcanzar tamaños muestrales más amplios, detectar efectos pequeños o poco frecuentes, y crear *cohortes poblacionales* de una magnitud inalcanzable para un único centro. En la actualidad, la puesta en marcha de la cohorte IMPaCT^14^, un proyecto de vanguardia liderado y desarrollado por profesionales de AP que seguirá a 200.000 personas durante 20 años, es uno de los proyectos nacionales de mayor impacto que permitirá comprender mejor el origen de las principales enfermedades en España y permitirá disponer de modelos predictivos para los principales problemas de salud, integrando información genética, individual y contextual con vistas a la prevención individualizada.

## Una cuestión de justicia ética y humanización

Investigar en AP es, ante todo, un ejercicio de *justicia ética*^15^^-^^17^. Nuestra cercanía nos permite dar voz y visibilizar a quienes sistemáticamente quedan fuera de los ensayos clínicos: niños y niñas, personas mayores, con demencia, con pluripatología, en cuidados paliativos o personas en situación de vulnerabilidad social o pertenecientes a minorías étnicas. Al integrarlos, no solo hacemos una ciencia más inclusiva, sino que validamos la evidencia previa en muestras que no son *idealizadas*, sino reales.

La AP tiene un recorrido de crecimiento considerable en líneas de investigación que le son especialmente propias^18^^,^^19^: la prevención, el autocuidado y el impacto de factores potencialmente salutogénicos; el efecto de los determinantes sociales en la salud y la investigación en salud en la familia y la comunidad; la investigación sobre multimorbilidad, cronicidad y polifarmacia en el entorno real del paciente; así como el impacto del vínculo terapéutico. La AP tiene un papel central en el desarrollo y la utilización de medidas de resultado percibidas por el paciente (PROMs), que capturan aquello que más importa a las personas (calidad de vida, funcionalidad, síntomas) y que con frecuencia escapa a los desenlaces puramente biomédicos que predominan en muchas de las investigaciones.

Además, la AP es el ámbito donde lo *invisible* cobra importancia. Algunas de las preguntas más relevantes en la AP no pueden responderse únicamente con cifras. Comprender la incertidumbre, la confianza, la adherencia, el sufrimiento o el impacto de la enfermedad en la vida cotidiana requiere metodologías capaces de explorar significados y experiencias. La investigación cualitativa aporta esa mirada complementaria, imprescindible para construir una evidencia que sea, además de sólida, clínicamente relevante y humanamente significativa. La AP es, probablemente, el ámbito donde las llamadas *capacidades blandas y cuidados invisibles*^20^^,^^21^ adquieren mayor relevancia clínica. La comunicación, la confianza, la continuidad asistencial, el acompañamiento o la gestión compartida de la incertidumbre no son únicamente valores profesionales: constituyen intervenciones complejas que influyen en la adherencia, la experiencia de enfermedad y los resultados en salud. Lejos de quedar al margen del conocimiento científico, representan un campo de investigación propio que requiere metodologías capaces de captar la complejidad de las relaciones humanas y del contexto en el que se desarrolla la atención.

## Claves para que la investigación en AP sea una realidad sostenible

Fomentar una investigación sólida en AP dependerá de la capacidad de crear un entorno que la haga posible: tiempo protegido, estructuras de apoyo, formación, colaboración con alianzas entre niveles asistenciales y reconocimiento del potencial liderazgo científico de sus profesionales.

* * *

En definitiva, apostar por la investigación en AP es impulsar una ciencia más útil, más aplicable, más cercana a la práctica clínica, más conectada con la realidad de las personas, y más próxima a las necesidades de la población.
